# Supplementary material for: Splice-Junction-Based Mapping of Alternative Isoforms in the Human Proteome
Source: Cell Rep. Author manuscript; Available in PMC 2020 Jan 15. (PMC6961840; doi:10.1016/j.celrep.2019.11.026)
Supplement: 2 [file NIHMS1546469-supplement-2.zip › DF1/PXD006675/RightVentricle/RightVentricle_26_TTN_SNCTVSVHVSEPATIVEKPESIK.pdf]

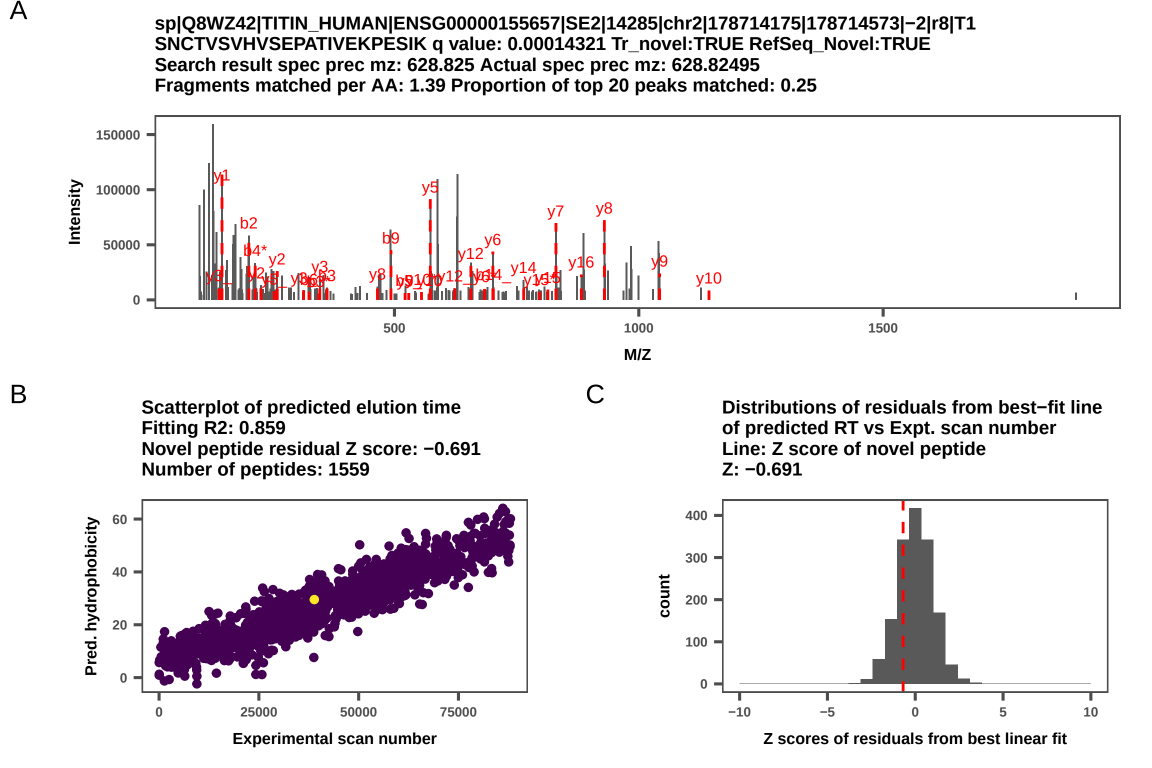

A
sp|Q8WZ42|TITIN_HUMAN|ENSG00000155657|SE2|14285|chr2|178714175|178714573|−2|r8|T1
SNCTVSVHVSEPATIVEKPESIK q value: 0.00014321 Tr_novel:TRUE RefSeq_Novel:TRUE
Search result spec prec mz: 628.825 Actual spec prec mz: 628.82495
Fragments matched per AA: 1.39 Proportion of top 20 peaks matched: 0.25
Intensity
150000
100000
50000
0
y1
b2
b4*
y2
y5
b9
y7
y8
y6
y12
y14
y16
y9
y10
500
1000
1500
M/Z
B
Scatterplot of predicted elution time
Fitting R2: 0.859
Novel peptide residual Z score: −0.691
Number of peptides: 1559
Pred. hydrophobicity
60
40
20
0
25000
50000
75000
Experimental scan number
C
Distributions of residuals from best−fit line
of predicted RT vs Expt. scan number
Line: Z score of novel peptide
Z: −0.691
count
400
300
200
100
−10
−5
5
10
Z scores of residuals from best linear fit
